# 4-[2-(3,4-Dimethoxy­phenethyl­amino)prop­oxy]-2-methoxy­benzamide

**DOI:** 10.1107/S1600536808015134

**Published:** 2008-05-24

**Authors:** Dian-Qing Wu, Hui-Bin Zhang, Bao-Min Xi

**Affiliations:** a Department of Medicinal Chemistry, College of Pharmacy, Southern Medical University, Guangzhou 510515, People’s Republic of China; b Center of Drug Discovery, China Pharmaceutical University, Nanjing 210009, People’s Republic of China

## Abstract

The title compound, C_21_H_28_N_2_O_5_, has two intra­molecular N—H⋯O hydrogen bonds. Inter­molecular N—H⋯O hydrogen bonds [graph-set motif *R*_2_^2^(8)] give rise to a dimer. Weak N—H⋯N hydrogen bonds between neighboring dimers further extend the crystal structure, which exhibits an infinite chain motif.

## Related literature

For related literature, see: Allen *et al.* (1987 ▶); Beduschi & Beduachi (1998 ▶); Bernstein *et al.*(1995 ▶); Boonnak *et al.* (2005 ▶); Gunderman *et al.* (1995 ▶); Hieble *et al.* (1995 ▶); Kasztreiner *et al.* (1989 ▶); Ng *et al.* (2005 ▶); Xi *et al.* (2005 ▶).
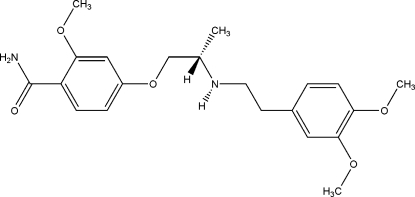


## Experimental

### 

#### Crystal data

C_21_H_28_N_2_O_5_
*M* *_r_* = 388.45
Monoclinic, 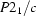

*a* = 7.7564 (3) Å
*b* = 9.3509 (4) Å
*c* = 29.9987 (12) Å
β = 95.370 (3)°
*V* = 2166.24 (15) Å^3^
*Z* = 4
Mo *K*α radiation
μ = 0.09 mm^−1^
*T* = 296 (2) K
0.21 × 0.18 × 0.17 mm

#### Data collection

Bruker APEXII area-detector diffractometer
Absorption correction: none
26019 measured reflections
3909 independent reflections
1966 reflections with *I* > 2σ(*I*)
*R*_int_ = 0.069

#### Refinement

*R*[*F* ^2^ > 2σ(*F* ^2^)] = 0.062
*wR*(*F* ^2^) = 0.183
*S* = 1.03
3909 reflections
260 parameters
1 restraint
H atoms treated by a mixture of independent and constrained refinement
Δρ_max_ = 0.24 e Å^−3^
Δρ_min_ = −0.22 e Å^−3^

### 

Data collection: *APEX2* (Bruker, 2004 ▶); cell refinement: *APEX2*; data reduction: *APEX2*; program(s) used to solve structure: *SHELXS97* (Sheldrick, 2008 ▶); program(s) used to refine structure: *SHELXL97* (Sheldrick, 2008 ▶); molecular graphics: *XP* in *SHELXTL* (Sheldrick, 2008 ▶); software used to prepare material for publication: *SHELXL97*.

## Supplementary Material

Crystal structure: contains datablocks I, global. DOI: 10.1107/S1600536808015134/zl2117sup1.cif

Structure factors: contains datablocks I. DOI: 10.1107/S1600536808015134/zl2117Isup2.hkl

Additional supplementary materials:  crystallographic information; 3D view; checkCIF report

## Figures and Tables

**Table 1:** Hydrogen-bond geometry (Å, °)

*D*—H⋯*A*	*D*—H	H⋯*A*	*D*⋯*A*	*D*—H⋯*A*
N2—H2*A*⋯O5^i^	0.86	2.05	2.909 (3)	178
N2—H2*B*⋯O4	0.86	2.04	2.684 (3)	131
N2—H2*B*⋯N1^ii^	0.86	2.54	3.161 (3)	129
N1—H1⋯O1	0.903 (17)	2.41 (3)	2.798 (3)	106 (2)

Symmetry codes: (i) 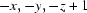; (ii) 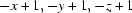.

**Fig. 1.**
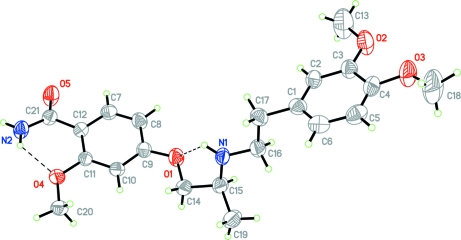
The molecular structure showing the atomic-numbering scheme. Displacement ellipsoids drawn at the 30% probability level. Intramolecular hydrogen bonds are shown as dashed lines.

**Fig. 2.**
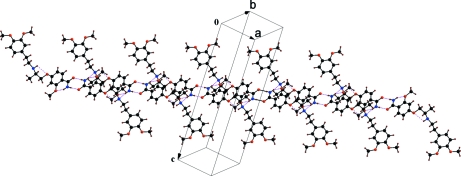
The molecular packing showing the intermolecular hydrogen bonding interactions as broken lines.

## supplementary crystallographic information

### Comment

α1-Adrenoreceptors (α1-AR) are members of the super family of seven transmembrane G protein coupled receptors (GPCR) (Gunderman *et al.*, 1995) and regulate several important physiological processes (Hieble *et al.*, 1995). In recent years, the search for new α1-adrenoreceptor antagonists has increased in parallel with the development of postsynaptically selective α-adrenoreceptor antagonists due to their importance in the treatment of hypertension (Kasztreiner *et al.*, 1989) and for prostatic hypertrophy (Beduschi & Beduachi, 1998). In the course of our studies on phenoxylalkylamine-phenylethanamine derivatives as potential antagonists of α1-adrenoreceptors, we have synthesized a library of compounds (Xi *et al. *, 2005) that show good activity. The title compound is one such phenoxylalkylamine-phenylethanamine derivative with α1-adrenoreceptor antagonist properties.

In the title compound (Fig. 1), the C—C bond lengths show normal values (Allen *et al.*, 1987), and the C—O and C=O bond lengths are comparable to those observed in simliar structures (Ng *et al.*, 2005; Boonnak *et al.*, 2005), while the C—N distances in the structure fall in the range of 1.308 (3)–1.469 (4) Å. The title molecular structure acts as hydrogen bonding donor and acceptor with two intramolecular N—H···O hydrogen bonds. The compound forms dimers with neighboring molecules through N—H···O hydrogen bonding with a *R*_2_^2^(8) graph set motif (Bernstein *et al.*, 1995), which are further self-assembled by N—H···N hydrogen bonds (table 1) to form an infinite chain (Fig. 2).

### Experimental

A mixture of 2-methoxy-4-(2-oxopropoxy)benzamide (0.4 g), 2-(3,4-dimethoxy- phenyl)ethanamine (0.4 ml), TsOH (3 drops), and methanol (20 ml) were heated to reflux of the solvent for 3 h. After cooling KBH_4_ (0.2 g) was added to the mixture portion wise over a period of 1 h and the mixture was stirred at room temperature for another 2 h. The methanol was evaporated, and water (15 ml) was added to the residue. The aqueous solution was extracted with ethyl acetate and the extract was dried over MgSO_4_, and evaporated. The residue was chromatographed on silica gel with petroleum ether and ethyl acetate (1:2 with triethylamine) as the eluent to obtain the colorless block crystals (0.3 g, 69.6%).

### Refinement

H atoms on carbon atoms and N2 were placed in calculated positions and were treated as riding on the parent C or N atoms with C—H = 0.92–0.97 Å and N—H = 0.86 Å. The H atom on N1 atom was tentatively located in a difference electron density Fourier map and was refined with distance restraint of N–H = 0.90 (2) Å. *U*_iso_(H) were set to 1.2 or 1.5 *U*_eq_(C) and 1.2 *U*_eq_(N).

### Crystal data

**Table:**

C_21_H_28_N_2_O_5_	*F*_000_ = 832
*M**_r_* = 388.45	*D*_x_ = 1.191 Mg m^−^^3^
Monoclinic, *P*2_1_/*c*	Mo *K*α radiation λ = 0.71073 Å
Hall symbol: -P 2ybc	Cell parameters from 2279 reflections
*a* = 7.7564 (3) Å	θ = 2.3–28.0º
*b* = 9.3509 (4) Å	µ = 0.09 mm^−^^1^
*c* = 29.9987 (12) Å	*T* = 296 (2) K
β = 95.370 (3)º	Block, colorless
*V* = 2166.24 (15) Å^3^	0.21 × 0.18 × 0.17 mm
*Z* = 4	

### Data collection

**Table:**

Bruker APEXII area-detector diffractometer	1966 reflections with *I* > 2σ(*I*)
Radiation source: fine-focus sealed tube	*R*_int_ = 0.069
Monochromator: graphite	θ_max_ = 25.2º
*T* = 296(2) K	θ_min_ = 2.3º
φ and ω scans	*h* = −8→9
Absorption correction: none	*k* = −11→11
26019 measured reflections	*l* = −35→35
3909 independent reflections	

### Refinement

**Table:**

Refinement on *F*^2^	Secondary atom site location: difference Fourier map
Least-squares matrix: full	Hydrogen site location: inferred from neighbouring sites
*R*[*F*^2^ > 2σ(*F*^2^)] = 0.062	H atoms treated by a mixture of independent and constrained refinement
*wR*(*F*^2^) = 0.183	*w* = 1/[σ^2^(*F*_o_^2^) + (0.0802*P*)^2^ + 0.288*P*] where *P* = (*F*_o_^2^ + 2*F*_c_^2^)/3
*S* = 1.03	(Δ/σ)_max_ < 0.001
3909 reflections	Δρ_max_ = 0.24 e Å^−^^3^
260 parameters	Δρ_min_ = −0.22 e Å^−^^3^
1 restraint	Extinction correction: none
Primary atom site location: structure-invariant direct methods	

### Special details

**Table:**

Geometry. All e.s.d.'s (except the e.s.d. in the dihedral angle between two l.s. planes) are estimated using the full covariance matrix. The cell e.s.d.'s are taken into account individually in the estimation of e.s.d.'s in distances, angles and torsion angles; correlations between e.s.d.'s in cell parameters are only used when they are defined by crystal symmetry. An approximate (isotropic) treatment of cell e.s.d.'s is used for estimating e.s.d.'s involving l.s. planes.
Refinement. Refinement of *F*^2^ against ALL reflections. The weighted *R*-factor *wR* and goodness of fit *S* are based on *F*^2^, conventional *R*-factors *R* are based on *F*, with *F* set to zero for negative *F*^2^. The threshold expression of *F*^2^ > σ(*F*^2^) is used only for calculating *R*-factors(gt) *etc*. and is not relevant to the choice of reflections for refinement. *R*-factors based on *F*^2^ are statistically about twice as large as those based on *F*, and *R*- factors based on ALL data will be even larger.

### Fractional atomic coordinates and isotropic or equivalent isotropic displacement parameters (Å^2^)

**Table:**

	*x*	*y*	*z*	*U*_iso_*/*U*_eq_	
C1	1.0849 (4)	0.9275 (3)	0.68939 (10)	0.0707 (8)	
C2	1.0775 (4)	0.8967 (3)	0.73403 (10)	0.0730 (9)	
H2	1.0012	0.8264	0.7419	0.088*	
C3	1.1788 (4)	0.9662 (3)	0.76742 (11)	0.0768 (9)	
C4	1.2923 (4)	1.0728 (3)	0.75616 (13)	0.0802 (9)	
C5	1.3002 (4)	1.1052 (4)	0.71190 (14)	0.0894 (10)	
H5	1.3752	1.1762	0.7038	0.107*	
C6	1.1968 (5)	1.0324 (4)	0.67897 (12)	0.0854 (10)	
H6	1.2040	1.0558	0.6491	0.102*	
C7	0.2048 (4)	0.3654 (3)	0.58051 (10)	0.0731 (9)	
H7	0.1768	0.2941	0.6002	0.088*	
C8	0.2697 (4)	0.4909 (4)	0.59783 (10)	0.0786 (9)	
H8	0.2876	0.5034	0.6287	0.094*	
C9	0.3086 (4)	0.5986 (3)	0.56951 (10)	0.0652 (8)	
C10	0.2844 (3)	0.5786 (3)	0.52359 (9)	0.0637 (8)	
H10	0.3093	0.6521	0.5043	0.076*	
C11	0.2230 (3)	0.4489 (3)	0.50650 (9)	0.0571 (7)	
C12	0.1785 (3)	0.3388 (3)	0.53488 (9)	0.0604 (7)	
C13	1.0809 (9)	0.8210 (6)	0.82400 (14)	0.194 (3)	
H13A	0.9600	0.8442	0.8191	0.292*	
H13B	1.1095	0.7984	0.8550	0.292*	
H13C	1.1054	0.7400	0.8059	0.292*	
C14	0.4230 (4)	0.8365 (3)	0.56233 (10)	0.0785 (9)	
H14A	0.3207	0.8859	0.5491	0.094*	
H14B	0.4874	0.8014	0.5384	0.094*	
C15	0.5335 (4)	0.9363 (3)	0.59209 (10)	0.0752 (9)	
H15	0.4726	0.9616	0.6182	0.090*	
C16	0.8086 (4)	0.9358 (3)	0.63960 (10)	0.0766 (9)	
H16A	0.7475	0.9578	0.6655	0.092*	
H16B	0.8422	1.0254	0.6266	0.092*	
C17	0.9687 (4)	0.8500 (4)	0.65416 (10)	0.0833 (10)	
H17A	1.0321	0.8313	0.6284	0.100*	
H17B	0.9348	0.7588	0.6660	0.100*	
C18	1.5228 (7)	1.2293 (6)	0.78212 (16)	0.171 (2)	
H18A	1.5999	1.1812	0.7638	0.257*	
H18B	1.5858	1.2594	0.8096	0.257*	
H18C	1.4738	1.3113	0.7664	0.257*	
C19	0.5701 (5)	1.0702 (3)	0.56651 (13)	0.1053 (12)	
H19A	0.6320	1.1371	0.5863	0.158*	
H19B	0.4628	1.1121	0.5544	0.158*	
H19C	0.6388	1.0465	0.5425	0.158*	
C20	0.2483 (4)	0.5301 (3)	0.43139 (9)	0.0771 (9)	
H20A	0.1776	0.6133	0.4343	0.116*	
H20B	0.2303	0.4945	0.4013	0.116*	
H20C	0.3679	0.5551	0.4381	0.116*	
C21	0.1018 (4)	0.1977 (3)	0.52065 (11)	0.0676 (8)	
N1	0.6938 (3)	0.8582 (2)	0.60697 (8)	0.0666 (7)	
H1	0.662 (4)	0.780 (2)	0.6217 (8)	0.080*	
N2	0.0991 (3)	0.1560 (3)	0.47879 (8)	0.0817 (8)	
H2A	0.0548	0.0747	0.4708	0.098*	
H2B	0.1417	0.2102	0.4594	0.098*	
O1	0.3744 (3)	0.7205 (2)	0.58938 (6)	0.0838 (7)	
O2	1.1798 (4)	0.9383 (3)	0.81226 (8)	0.1191 (10)	
O3	1.3892 (3)	1.1353 (3)	0.79165 (9)	0.1160 (9)	
O4	0.2023 (3)	0.4228 (2)	0.46171 (6)	0.0753 (6)	
O5	0.0413 (4)	0.1217 (2)	0.54918 (8)	0.1049 (9)	

### Atomic displacement parameters (Å^2^)

**Table:**

	*U*^11^	*U*^22^	*U*^33^	*U*^12^	*U*^13^	*U*^23^
C1	0.063 (2)	0.075 (2)	0.073 (2)	0.0073 (17)	0.0014 (16)	0.0039 (17)
C2	0.065 (2)	0.071 (2)	0.082 (2)	−0.0139 (16)	0.0032 (16)	−0.0044 (17)
C3	0.079 (2)	0.076 (2)	0.076 (2)	−0.0129 (19)	0.0067 (18)	−0.0039 (17)
C4	0.072 (2)	0.070 (2)	0.099 (3)	−0.0126 (18)	0.0079 (19)	−0.011 (2)
C5	0.073 (2)	0.077 (2)	0.120 (3)	−0.0097 (19)	0.019 (2)	0.015 (2)
C6	0.081 (2)	0.090 (3)	0.086 (2)	0.011 (2)	0.012 (2)	0.018 (2)
C7	0.082 (2)	0.070 (2)	0.069 (2)	−0.0096 (18)	0.0146 (16)	0.0086 (16)
C8	0.091 (2)	0.083 (2)	0.0625 (18)	−0.0174 (19)	0.0095 (17)	0.0007 (18)
C9	0.0610 (19)	0.0651 (19)	0.069 (2)	−0.0088 (15)	0.0022 (15)	−0.0018 (16)
C10	0.0598 (19)	0.0609 (18)	0.069 (2)	−0.0022 (15)	−0.0005 (14)	0.0091 (15)
C11	0.0533 (17)	0.0567 (17)	0.0608 (18)	0.0012 (14)	0.0018 (13)	0.0001 (14)
C12	0.0518 (17)	0.0616 (18)	0.0688 (19)	−0.0031 (14)	0.0103 (14)	0.0015 (15)
C13	0.327 (8)	0.172 (5)	0.088 (3)	−0.131 (6)	0.039 (4)	−0.003 (3)
C14	0.081 (2)	0.066 (2)	0.085 (2)	−0.0101 (17)	−0.0076 (17)	0.0068 (17)
C15	0.069 (2)	0.0654 (19)	0.088 (2)	−0.0055 (17)	−0.0093 (17)	0.0026 (17)
C16	0.074 (2)	0.075 (2)	0.079 (2)	−0.0001 (17)	−0.0057 (17)	−0.0088 (16)
C17	0.083 (2)	0.087 (2)	0.077 (2)	0.0133 (19)	−0.0053 (18)	−0.0072 (17)
C18	0.145 (4)	0.178 (5)	0.192 (5)	−0.105 (4)	0.023 (4)	−0.035 (4)
C19	0.101 (3)	0.071 (2)	0.137 (3)	−0.008 (2)	−0.022 (2)	0.009 (2)
C20	0.089 (2)	0.073 (2)	0.0697 (19)	0.0018 (18)	0.0097 (17)	0.0126 (16)
C21	0.067 (2)	0.0626 (19)	0.074 (2)	−0.0052 (16)	0.0160 (16)	0.0004 (17)
N1	0.0687 (17)	0.0610 (15)	0.0689 (15)	−0.0033 (13)	0.0005 (13)	−0.0027 (12)
N2	0.101 (2)	0.0681 (16)	0.0786 (18)	−0.0248 (15)	0.0219 (15)	−0.0060 (14)
O1	0.0948 (17)	0.0803 (15)	0.0759 (14)	−0.0244 (13)	0.0059 (12)	−0.0059 (12)
O2	0.157 (2)	0.126 (2)	0.0737 (16)	−0.0677 (19)	0.0075 (15)	−0.0092 (14)
O3	0.1067 (19)	0.113 (2)	0.128 (2)	−0.0478 (17)	0.0056 (16)	−0.0250 (16)
O4	0.0972 (16)	0.0654 (13)	0.0627 (13)	−0.0120 (11)	0.0038 (11)	0.0065 (10)
O5	0.147 (2)	0.0836 (16)	0.0905 (16)	−0.0453 (16)	0.0465 (15)	−0.0088 (13)

### Geometric parameters (Å, °)

**Table:**

C1—C6	1.365 (4)	C14—C15	1.503 (4)
C1—C2	1.376 (4)	C14—H14A	0.9700
C1—C17	1.509 (4)	C14—H14B	0.9700
C2—C3	1.377 (4)	C15—N1	1.475 (4)
C2—H2	0.9300	C15—C19	1.509 (4)
C3—O2	1.369 (4)	C15—H15	0.9800
C3—C4	1.393 (4)	C16—N1	1.454 (3)
C4—C5	1.369 (4)	C16—C17	1.508 (4)
C4—O3	1.375 (4)	C16—H16A	0.9700
C5—C6	1.391 (5)	C16—H16B	0.9700
C5—H5	0.9300	C17—H17A	0.9700
C6—H6	0.9300	C17—H17B	0.9700
C7—C8	1.361 (4)	C18—O3	1.408 (4)
C7—C12	1.387 (4)	C18—H18A	0.9600
C7—H7	0.9300	C18—H18B	0.9600
C8—C9	1.369 (4)	C18—H18C	0.9600
C8—H8	0.9300	C19—H19A	0.9600
C9—O1	1.363 (3)	C19—H19B	0.9600
C9—C10	1.385 (4)	C19—H19C	0.9600
C10—C11	1.383 (4)	C20—O4	1.422 (3)
C10—H10	0.9300	C20—H20A	0.9600
C11—O4	1.360 (3)	C20—H20B	0.9600
C11—C12	1.400 (4)	C20—H20C	0.9600
C12—C21	1.493 (4)	C21—O5	1.238 (3)
C13—O2	1.403 (5)	C21—N2	1.313 (3)
C13—H13A	0.9600	N1—H1	0.903 (17)
C13—H13B	0.9600	N2—H2A	0.8600
C13—H13C	0.9600	N2—H2B	0.8600
C14—O1	1.426 (3)		
			
C6—C1—C2	117.3 (3)	N1—C15—C19	111.8 (3)
C6—C1—C17	122.4 (3)	C14—C15—C19	109.7 (3)
C2—C1—C17	120.3 (3)	N1—C15—H15	109.5
C1—C2—C3	122.4 (3)	C14—C15—H15	109.5
C1—C2—H2	118.8	C19—C15—H15	109.5
C3—C2—H2	118.8	N1—C16—C17	111.4 (2)
O2—C3—C2	125.1 (3)	N1—C16—H16A	109.3
O2—C3—C4	115.4 (3)	C17—C16—H16A	109.3
C2—C3—C4	119.5 (3)	N1—C16—H16B	109.3
C5—C4—O3	125.9 (3)	C17—C16—H16B	109.3
C5—C4—C3	118.7 (3)	H16A—C16—H16B	108.0
O3—C4—C3	115.4 (3)	C16—C17—C1	111.6 (3)
C4—C5—C6	120.4 (3)	C16—C17—H17A	109.3
C4—C5—H5	119.8	C1—C17—H17A	109.3
C6—C5—H5	119.8	C16—C17—H17B	109.3
C1—C6—C5	121.7 (3)	C1—C17—H17B	109.3
C1—C6—H6	119.1	H17A—C17—H17B	108.0
C5—C6—H6	119.1	O3—C18—H18A	109.5
C8—C7—C12	123.2 (3)	O3—C18—H18B	109.5
C8—C7—H7	118.4	H18A—C18—H18B	109.5
C12—C7—H7	118.4	O3—C18—H18C	109.5
C7—C8—C9	119.5 (3)	H18A—C18—H18C	109.5
C7—C8—H8	120.3	H18B—C18—H18C	109.5
C9—C8—H8	120.3	C15—C19—H19A	109.5
O1—C9—C8	116.0 (3)	C15—C19—H19B	109.5
O1—C9—C10	123.9 (3)	H19A—C19—H19B	109.5
C8—C9—C10	120.0 (3)	C15—C19—H19C	109.5
C11—C10—C9	119.8 (3)	H19A—C19—H19C	109.5
C11—C10—H10	120.1	H19B—C19—H19C	109.5
C9—C10—H10	120.1	O4—C20—H20A	109.5
O4—C11—C10	121.9 (2)	O4—C20—H20B	109.5
O4—C11—C12	117.1 (2)	H20A—C20—H20B	109.5
C10—C11—C12	121.0 (3)	O4—C20—H20C	109.5
C7—C12—C11	116.5 (3)	H20A—C20—H20C	109.5
C7—C12—C21	117.3 (3)	H20B—C20—H20C	109.5
C11—C12—C21	126.2 (3)	O5—C21—N2	121.2 (3)
O2—C13—H13A	109.5	O5—C21—C12	118.4 (3)
O2—C13—H13B	109.5	N2—C21—C12	120.4 (3)
H13A—C13—H13B	109.5	C16—N1—C15	113.7 (2)
O2—C13—H13C	109.5	C16—N1—H1	104.8 (18)
H13A—C13—H13C	109.5	C15—N1—H1	106.9 (19)
H13B—C13—H13C	109.5	C21—N2—H2A	120.0
O1—C14—C15	107.5 (2)	C21—N2—H2B	120.0
O1—C14—H14A	110.2	H2A—N2—H2B	120.0
C15—C14—H14A	110.2	C9—O1—C14	119.7 (2)
O1—C14—H14B	110.2	C3—O2—C13	116.3 (3)
C15—C14—H14B	110.2	C4—O3—C18	117.9 (3)
H14A—C14—H14B	108.5	C11—O4—C20	119.4 (2)
N1—C15—C14	106.8 (2)		

### Hydrogen-bond geometry (Å, °)

**Table:**

*D*—H···*A*	*D*—H	H···*A*	*D*···*A*	*D*—H···*A*
N2—H2A···O5^i^	0.86	2.05	2.909 (3)	178
N2—H2B···O4	0.86	2.04	2.684 (3)	131
N2—H2B···N1^ii^	0.86	2.54	3.161 (3)	129
N1—H1···O1	0.903 (17)	2.41 (3)	2.798 (3)	106 (2)

Symmetry codes: (i) −*x*, −*y*, −*z*+1; (ii) −*x*+1, −*y*+1, −*z*+1.
